# A rare case of multi-segmental lumbar intradural ependymoma: clinical presentation and surgical management: a case report

**DOI:** 10.3389/fonc.2026.1906737

**Published:** 2026-08-21

**Authors:** Tao Chen, Jianfeng Chen, Bin Wu, Weijin Li, Fan Zhang, Jie Lin

**Affiliations:** 1 The First Clinical Medical College of China Three Gorges University, Yichang, Hubei, China; 2 Spinal Surgery Department, Yichang Central People’s Hospital, Yichang, China; 3 The Second People’s Hospital of China Three Gorges University, Yichang, Hubei, China

**Keywords:** case report, ependymoma, lumbar, multi-segment lumbar, spine

## Abstract

**Background:**

Intradural extramedullary(IDEM)-type Ependymoma is a rare clinicalentity belonging to the broader category of uncommon glial neoplasms. To date, most published studies have focused predominantly on classic anatomical locations—particularly the filum terminale, whereas the present case arose from an atypical spinal site, highlighting the pronounced anatomical heterogeneity characteristic of this tumor subtype. Moreover, its neuroimaging features demonstrate considerable overlap with those of more common intraspinal tumors, including schwannomas and meningiomas, thereby presenting a substantial diagnostic challenge and elevating the risk of misdiagnosis.increasing the risk of misdiagnosis.

**Case presentation:**

30-year-old male patient underwent contrast-enhanced lumbar MRI, which revealed a hypervascular intradural tumor spanning the L1-L3 vertebral levels. Complete surgical resection was achieved; the resected specimen measured 4.5 cm×2.5 cm×1.0 cm. Postoperative histopathological examination confirmed the diagnosis of spinal ependymoma. Spinal ependymomas most commonly arise in the filum terminale region, where they are typically amenable to gross-total resection with a relatively low risk of permanent neurological deficit. In this case, however, the tumor was located at the L1-L3 level,a region densely populated by motor neurons and autonomic nerve fibers innervating the lower limbs and pelvic viscera. Consequently, the tumor posed a heightened risk of infiltration into adjacent nerve roots or spinal cord parenchyma. Differential diagnosis:(1) Schwannoma. On MRI, schwannomas are typically iso- to hypointense on T1-weighted images and hyperintense on T2-weighted images. Cystic degeneration is common, and contrast-enhanced imaging generally demonstrates enhancement of the solid component, particularly in Antoni A–predominant areas, whereas cystic regions remain non-enhancing. Intradural extramedullary (IDEM) ependymomas may likewise appear iso- to hypointense on T1-weighted images and iso- to hyperintense on T2-weighted images; however, cystic change, hemorrhage, and heterogeneous enhancement of the solid component may be more prominent. Both lesions can produce neural compression and pain, although radicular pain is more characteristic of schwannoma because of its frequent origin from a spinal nerve root.(2) Spinal meningioma. Spinal meningiomas occur predominantly in middle-aged women and usually appear as well-circumscribed round, oval, or plaque-like intradural extramedullary masses. They are commonly isointense on both T1- and T2-weighted images and demonstrate strong, usually homogeneous enhancement, often accompanied by a dural tail sign. Clinical manifestations may include localized back pain, radicular pain, gait disturbance, and sensory impairment. In contrast, IDEM ependymomas arising in the conus medullaris–cauda equina region may present with more diffuse pain, bilateral lower-extremity weakness, sensory disturbances, or sphincter dysfunction, including urinary retention.(3) Lipoma.Spinal lipomas typically demonstrate homogeneous high signal intensity on T1-weighted images, with complete signal suppression on fat-saturated sequences and little or no contrast enhancement. They are more frequently associated with congenital spinal abnormalities and are commonly detected in children or young patients.(4) Metastatic disease. Spinal ependymomas are generally solitary intradural lesions, often involving the conus medullaris, filum terminale, or cauda equina region, and usually show enhancement of the solid tumor component. By contrast, spinal metastases occur more commonly in older patients and are often associated with a known primary malignancy. Imaging may demonstrate multifocal or noncontiguous vertebral marrow replacement, low signal intensity on T1-weighted images, contrast enhancement, cortical destruction, and possible epidural or paraspinal extension. Nevertheless, intradural metastasis should also be considered when a patient has a history of systemic cancer or evidence of disseminated disease.

**Conclusions:**

This case report describes a rare multi-segmental lumbar intradural extramedullary ependymoma located at the atypical L1–L3 spinal levels, emphasizing the diagnostic and therapeutic challenges associated with non-classical anatomical presentations. Unlike the more commonly reported filum terminale ependymomas, tumors arising in the upper lumbar region are closely related to cauda equina nerve roots, motor pathways, and autonomic neural structures, thereby requiring a surgical strategy that balances maximal tumor resection with preservation of neurological function. In line with current international perspectives, contrast-enhanced MRI should be regarded as the first-line modality for preoperative localization, assessment of tumor enhancement patterns, and evaluation of its relationship with adjacent neural structures; however, definitive diagnosis still depends on integrated histopathological, immunohistochemical, and molecular evaluation. In the present case, gross-total resection was achieved with preservation of neurological function, and no evidence of recurrence was observed during follow-up, supporting the view that surgery remains the cornerstone of treatment for low-grade spinal ependymoma, while adjuvant radiotherapy should be considered mainly in cases of subtotal resection, residual disease, recurrence, or higher-grade pathology. More importantly, this case highlights the need to overcome location-based diagnostic bias, include ependymoma in the differential diagnosis of atypical lumbar intradural extramedullary tumors, and adopt a multidisciplinary, function-preserving, precision-based management strategy. Long-term radiological surveillance remains essential because recurrence may occur even after apparently complete resection. Therefore, this report provides clinically relevant evidence for improving recognition, individualized surgical planning, and postoperative follow-up strategies for rare lumbar IDEM ependymomas.

## Introduction

IDEM ependymomas account for less than 5% of all spinal cord tumors. Lesions occurring at this atypical location pose unique diagnostic challenges, as their imaging characteristics frequently overlap with those of more common intradural extramedullary tumors-such as schwannomas and meningiomas-thereby predisposing to misdiagnosis. Recent reports on spinal ependymoma in the past 5 years (**Table 1**). Therefore, intraoperative neuroprotection-emphasizing preservation of neural architecture and functional integrity-was paramount.

**Table 1:** Literature review of recently published spinal ependymoma cases (2021-2025).

First author and year	Sex/age	Tumor location	Pathology	Therapy	Radiotherapy details	Follow-up	Recurrence/outcome
Zhang/Mirbolook, 2021/2023 (19, 20)	M/42	Oblongata to T4	Intramedullary ependymoma	Laminectomy from occiput to T4; resection	NR	NR	NR
Makino, 2021 (3)	NR	Intradural extramedullary spinal location	Ependymoma	Total resection	NR	>3 yrs reported	No
Trivedi, 2021 (21)	F/27	IDEM, D11-S1; conus attachment	WHO grade 2 non-myxopapillary ependymoma	D10-S1 laminectomy + surgical resection	NR	6 months	No
Atoot, 2021 (22)	M/43	Cervical intramedullary; C4-C7 syrinx, C7-T1 lesion	Intramedullary ependymoma	Diagnostic work-up; neurosurgical management discussed	NR	NR	NR
D'Angiolillo, 2021 (23)	M/45	Lumbosacral / cauda equina region	Myxopapillary ependymoma	NR	NR	NR	NR
Shields, 2022 (24)	NR	Multifocal intradural extramedullary spinal lesions	MYCN-amplified ependymoma	Tumor resection	Craniospinal irradiation reported	NR	Aggressive subtype
Almatrafi, 2023 (25)	M/28	Multicentric conus/cauda/filum region	Myxopapillary ependymoma	Surgical management reported	NR	NR	NR
Soto, 2023 (26)	M/50	Lumbosacral / filum terminale region	Myxopapillary ependymoma	Surgical management reviewed	NR	Close follow-up recommended	NR
Choi, 2023 (27)	F/43	Intradural extramedullary spinal lesion	Ependymoma with hemorrhage	NR	NR	NR	NR
Balodis, 2024 (28)	M/44	IDEM L1-S4; sacral erosion; neural-axis metastases	Myxopapillary ependymoma, CNS WHO grade 2	Biopsy + partial resection; laminectomy/laminoplasty	Adjuvant RT	1 year	Yes; growth/metastases
Kouhen, 2024 (29)	NR/68	Sacrum	Myxopapillary ependymoma	Sacral laminectomy + subtotal excision	Adjuvant RT	NR	Favorable outcome
Oke, 2025 (30)	NR	Lumbosacral spine	Myxopapillary ependymoma, CNS grade 2	Complete surgical removal	NR	3 months	No residual tumor
Arjomand, 2025 (31)	NR	Filum terminale / lumbosacral region	Myxopapillary ependymoma	NR	NR	NR	NR
Yuan, 2025 (32)	NR	Intradural extramedullary spinal lesion	Ependymoma + meningioma double primary	Surgical treatment reported	NR	NR	NR

IDEM, intradural extramedullary; MPE, myxopapillary ependymoma; RT, radiotherapy; NR, not reported in accessible abstract/full text.

This table is a literature-style summary made from recent accessible case reports/case-report abstracts; for SCI revision, verify each full text before submission.

Representative sources include Cureus, NMC Case Rep J, J Neurosurg Case Lessons, Radiology Case Reports, American Journal of Case Reports, and Surgical Neurology International.

**Figure 1:**
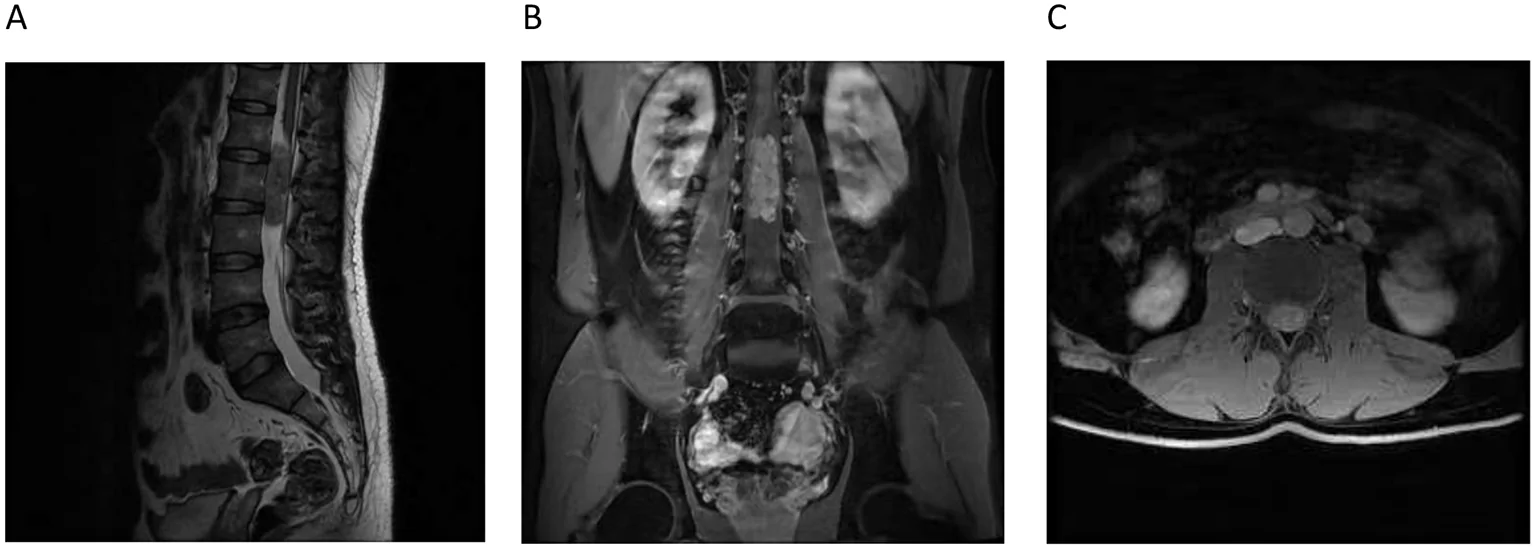
Lumbar spinal MRI findings in the patient. **(A)** Contrast-enhanced sagittal view; **(B)** Contrast-enhanced coronal view; **(C)** Contrast-enhanced axial view. A fusiform intradural tumor, measuring approximately 15 mm × 18 mm × 60 mm, is identified within the spinal canal at the L1–L3 vertebral levels. The lesion exhibits heterogeneous contrast enhancement. The cauda equina is compressed and displaced to the right; additionally, multiple tortuous vessels are observed adjacent to the spinal cord above the lesion.

This case highlights a critical diagnostic and therapeutic dilemma: the filum terminale is a vestigial, non-functional neural structure; consequently, tumors arising here are typically amenable to complete resection with minimal risk of neurological morbidity. In contrast, the L1-L3 spinal segments harbor autonomic centers and motor neuron cell bodies (**Figure 1**); tumors in this region often intimately envelop nerve roots or infiltrate the spinal cord parenchyma. This fundamental anatomical distinction constitutes a core clinical pitfall-namely, that for tumors located at atypical spinal levels, the surgical paradigm must shift from an uncompromising pursuit of “gross-total resection” toward a nuanced, function-preserving balance between maximal safe resection and preservation of critical neurological structures. This case underscores three essential clinical lessons:

1. Overcoming location-based diagnostic bias: When evaluating intradural spinal masses, clinicians must transcend the conventional association of filum terminale with IDEM ependymoma and systematically consider the entire spinal axis in the differential diagnosis.
2. Relying on multimodal assessment: Contrast-enhanced MRI remains the cornerstone for evaluating tumor vascularity, relationships with adjacent neural and vascular structures, and degree of spinal cord compression. However, definitive diagnosis and molecular subclassification ultimately depend on postoperative histopathological examination and immunohistochemical profiling; integration of both modalities is indispensable to prevent diagnostic error.
3. Prioritizing functional preservation in decision-making: For IDEM ependymomas involving neurologically critical segments—such as L1-L3,the primary surgical objective must be carefully calibrated between oncological completeness and maximal preservation of spinal cord and nerve root function. Intraoperative neurophysiological monitoring and microsurgical techniques are therefore indispensable. Although this patient was successfully managed via lumbar laminectomy, tumor resection, and spinal instrumentation, the clinical course profoundly illustrates that rare IDEM ependymomas arising at atypical spinal levels demand heightened vigilance and a multidisciplinary, individualized, precision-based diagnostic and therapeutic strategy to optimize long-term neurological and oncological outcomes.

## Case presentation

**Figure 2:**
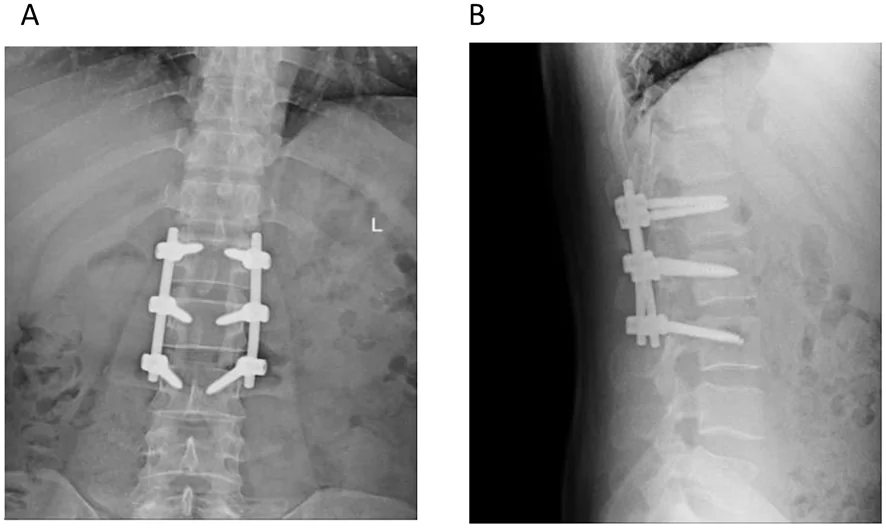
Radiographic follow-up findings at two weeks postoperatively **(A, B)** Anteroposterior and lateral lumbar spine radiographs demonstrate preserved natural lumbar lordosis and orderly vertebral alignment; mild osteophytic changes are observed in several vertebral bodies and associated posterior elements; no significant spinal canal stenosis is present.

**Figure 3:**
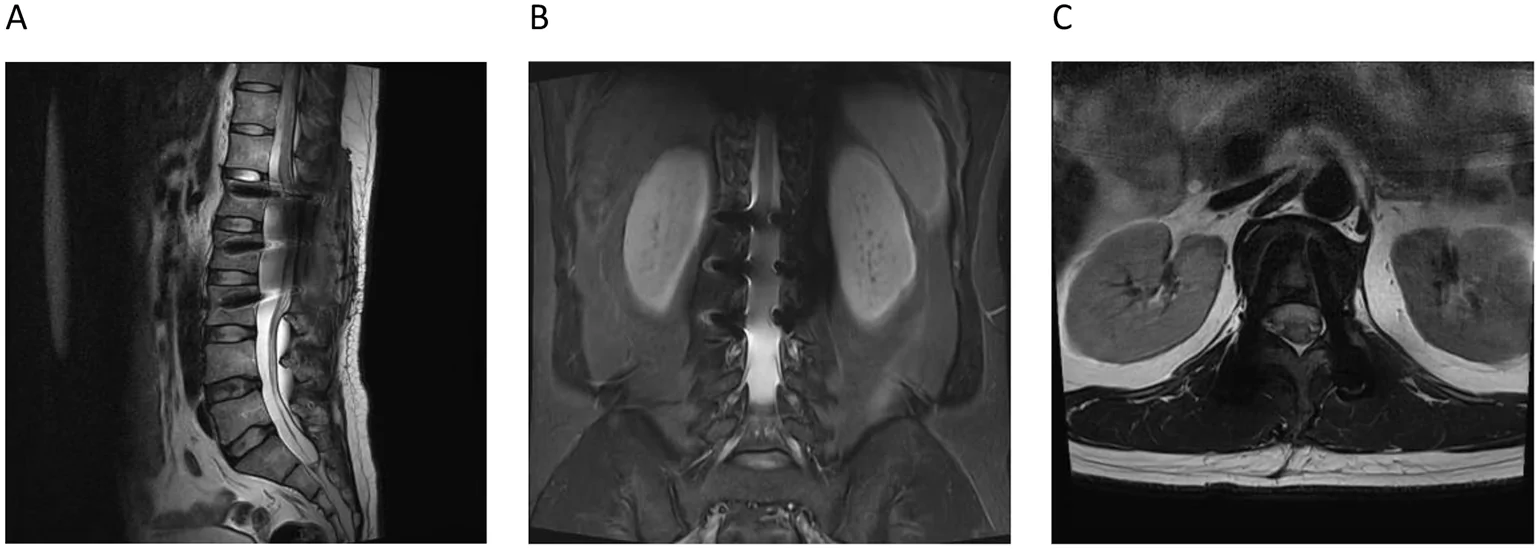
MRI contrast-enhanced follow-up examination at six months postoperatively. **(A)** Contrast-enhanced sagittal view; **(B)** Contrast-enhanced coronal view; **(C)** Contrast-enhanced axial view showing no obvious abnormal enhancement. Degenerative changes and bulging are observed at the L4/L5 and L5/S1 intervertebral discs.

**Figure 4:**
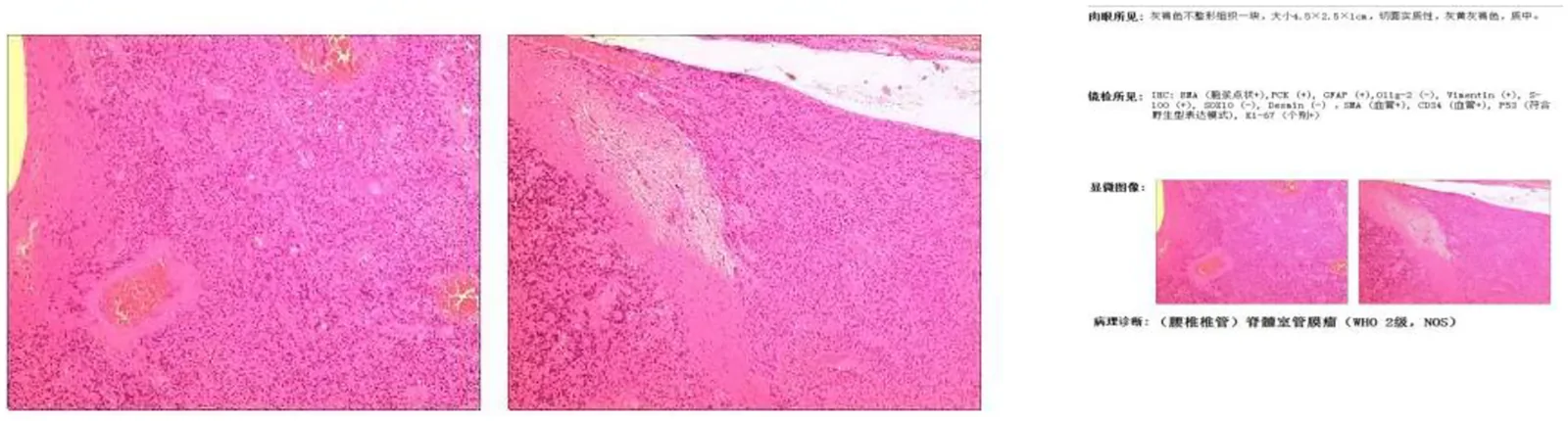
Postoperative pathological examination result (lumbar spinal canal): Spinal cord ependymoma (WHO grade 2, NOS). Microscopic findings:IHC: EMA (cytoplasmic dot-like positive), pan-cytokeratin (CK, positive), GFAP (positive), Olig-2 (negative), vimentin (positive), S-100 (positive), SOX10 (negative), desmin (negative), SMA (positive in vascular smooth muscle), CD34 (positive in vascular endothelium), p53 (expression pattern consistent with wild-type), Ki-67 (sporadic positivity).

A 30-year-old male administrative clerk presented with 5 days of persistent, non-radiating dull lumbosacral pain. Onset was insidious, with no trigger or prior treatment. He had no history of genetic, psychiatric, or chronic systemic disease, surgery, or trauma. Vital signs were stable on presentation. Neurological exam was normal. Neurological examination revealed full motor strength in both lower extremities (Medical Research Council grade 5/5) with normal muscle tone. Sensation was intact bilaterally in the lower extremities, including the saddle and perineal regions. Patellar and Achilles tendon reflexes were symmetrically elicited, and no pathological reflexes were present. Gait was normal, bowel and bladder function was preserved, and bilateral straight-leg-raising tests were negative. Physical examination revealed localized tenderness and percussion tenderness over the T12-L2 spinous processes, mild restriction of lumbar range of motion, and intact bilateral lower extremity neurological function. Day 0: Patient presented with 5 days of persistent low back pain. Preoperative imaging and evaluation conducted. Surgical intervention performed. Postoperative recovery monitored bodies (**Figures 2**, **3**). 6-month follow-up: contrast-enhanced MRI performed.1-year follow-up: phone consultation conducted. Contrast-enhanced lumbar MRI demonstrated an intradural, extramedullary, fusiform, heterogeneously enhancing mass extending from L1 to L3, resulting in compression and displacement of the cauda equina. Preoperative MRI findings were differentiated from schwannomas (target sign) and meningiomas (dural tail sign). Histopathological and molecular analysis confirmed a diagnosis of spinal ependymoma (World Health Organization grade 2), characterized predominantly by somatic NF2 mutations or monosomy 22/22q copy number loss. The patient underwent laminectomy at L1–L3, complete resection of the intradural tumor, and posterior spinal fusion with pedicle screw instrumentation. Adjuvant radiotherapy, chemotherapy, targeted therapy, or immunotherapy was not administered (**Figure 4**). At the 6-month follow-up, contrast-enhanced MRI showed no residual or recurrent enhancing lesion at the surgical site. One year after discharge, due to inconvenience in seeking medical treatment, the patient was followed up by phone. The patient reported no discomfort and no neurological dysfunction. The quality of life was satisfactory. Routine surveillance with serial neuroimaging and clinical neurological assessment is recommended.The distinctiveness of this case lies not only in the location of the lesion at the L1–L3 vertebral levels, but also in its anatomical relationships, operative management, and histopathological phenotype, all of which differ from those of the more common filum terminale–derived ependymomas. Typical tumors arising from the filum terminale are predominantly myxopapillary ependymomas, which usually have an identifiable attachment to the filum and are characterized histologically by papillary arrangements of tumor cells around hyalinized fibrovascular cores with a perivascular myxoid matrix. In contrast, the present tumor was located entirely within the intradural extramedullary compartment, with its cranial pole closely abutting the inferior aspect of the conus medullaris and its main bulk occupying the cauda equina region, where it compressed and displaced multiple nerve roots. No definite attachment to the filum terminale or origin from an individual cauda equina nerve root was identified intraoperatively. Because there was no non-functional filum-based stalk that could be safely divided, resection required intracapsular debulking under intraoperative neurophysiological monitoring, followed by meticulous extracapsular dissection between the functional cauda equina nerve roots and the traversing and feeding vessels.

This operative complexity was greater than that typically encountered in tumors with a clearly defined filum terminale attachment. Histologically, the tumor exhibited characteristic perivascular pseudorosettes, dot-like cytoplasmic EMA immunoreactivity, and GFAP positivity, without myxopapillary architecture, supporting the diagnosis of a conventional spinal ependymoma rather than a myxopapillary ependymoma. Accordingly, the combination of a purely intradural extramedullary growth pattern, absence of a definite filum terminale or nerve-root attachment, and a non-myxopapillary histological phenotype makes this lesion considerably rarer than both typical filum terminale–derived ependymomas and conventional intramedullary spinal ependymomas.

## Discussion

IDEM ependymoma is a clinically rare and uncommon glial tumor. Based on histopathological features, molecular characteristics, and anatomical location, it is classified into three subtypes: supratentorial, posterior fossa, and spinal. The severity of clinical symptoms correlates closely with the spinal cord segment involved by the intradural lesion (1). Radiological findings often overlap with those of more common tumors, such as schwannoma and meningioma-leading topotential diagnostic pitfalls. This patient did not have a comprehensive understanding of the disease, which resulted in the need for diagnosis after visiting two hospitals during the treatment process. During the treatment, everything went smoothly and no other complications occurred. The typical location is the filum terminale region caudal to the conus medullaris, a vestigial, non-functional membranous structure; thus, complete surgical resection can usually be achieved without neurologic deficit. In contrast, the L1–L3 spinal segments contain autonomic centers and motor neuron cell bodies; tumors in this region frequently envelop nerve roots or infiltrate the spinal cord parenchyma, necessitating meticulous preservation of critical neural structures during surgery.

Under general anesthesia, the patient underwent posterior lumbar laminectomy, resection of an intradural extramedullary tumor, and pedicle-screw fixation. The patient was placed in the prone position, and a radiopaque skin marker was applied. Intraoperative C-arm fluoroscopy was used to identify and confirm the L1–L3 vertebral levels. Based on the location and longitudinal extent of the lesion, an approximately 10-cm posterior midline lumbar incision was made. After exposure of the posterior elements at the relevant levels, six polyaxial pedicle screws measuring 45–65 mm in length were inserted bilaterally at L1–L3.Bony decompression was subsequently performed using an ultrasonic bone scalpel. The L2 lamina was completely removed, together with partial resection of the inferior portion of the L1 lamina and the superior portion of the L3 lamina, to ensure adequate exposure of the cranial and caudal poles of the tumor. The ultrasonic bone scalpel was operated at a frequency of 30 kHz and an amplitude of 60 μm under continuous saline irrigation for cooling. The laminar cortex was divided in a controlled manner; when approaching the ligamentum flavum and dura mater, the power output was reduced and blunt dissection was used to minimize mechanical and thermal injury to the dura and adjacent neural structures.Following laminectomy, the dura mater and arachnoid membrane were sequentially opened under an operating microscope, and the exposed neural structures were protected with moist cottonoid patties. Intraoperative exploration revealed an intradural extramedullary tumor extending from the L1 to L3 vertebral levels. Its cranial pole closely abutted the inferior aspect of the conus medullaris, whereas the main tumor bulk occupied the cauda equina compartment, resulting in marked compression and rightward displacement of the cauda equina nerve roots. The tumor was well encapsulated and surrounded by traversing vessels and vessels supplying the adjacent neural structures. Adhesive interfaces were present between the tumor and the adjacent cauda equina nerve roots and neural structures at the inferior aspect of the conus medullaris. No definite attachment or continuity between the tumor and the filum terminale was identified, and no clear origin from an individual cauda equina nerve root was observed.

Under intraoperative neurophysiological monitoring, the tumor capsule was initially opened, and piecemeal intracapsular debulking was performed to reduce the tumor volume and minimize traction on the conus medullaris and cauda equina nerve roots. The tumor was then progressively mobilized along the extracapsular dissection plane using a combination of blunt and sharp microsurgical techniques. Low-power bipolar coagulation was applied to the adhesive interfaces and peritumoral vessels. Particular attention was paid to preserving the cauda equina nerve roots, neural feeding vessels, and other traversing vascular structures while avoiding excessive traction, thermal injury, and ischemic neural damage. Because the tumor extended across multiple vertebral levels and was closely associated with the surrounding nerve roots and vascular structures, microsurgical dissection was technically demanding. Nevertheless, the relatively intact capsule provided an identifiable extracapsular plane, allowing gross-total resection to be achieved.The resected specimen consisted of an irregular, gray-brown, solid tissue mass measuring approximately 4.5 × 2.5 × 1.0 cm, with a gray-yellow to gray-brown cut surface.

After tumor removal, meticulous hemostasis was achieved, and the anatomical integrity of the inferior conus medullaris and cauda equina nerve roots was confirmed. The dura was then closed in a watertight fashion using nonabsorbable sutures. A Valsalva maneuver performed after dural closure revealed no evidence of cerebrospinal fluid leakage. The paraspinal muscles, deep fascia, subcutaneous tissue, and skin were subsequently closed in layers. Intraoperative exploration revealed an intradural extramedullary tumor extending from the L1 to L3 vertebral levels. Its cranial pole closely abutted the inferior aspect of the conus medullaris, while the main tumor bulk occupied the cauda equina compartment, resulting in compression and rightward displacement of the cauda equina nerve roots. No definite attachment or continuity between the tumor and the filum terminale was identified intraoperatively.

Immediate postoperative neurological examination showed stable neurological function without any new deficits. The patient reported marked relief of low-back pain, with no new or worsening radicular pain, numbness, or other symptoms suggestive of nerve-root dysfunction. Motor and sensory function in both lower extremities remained intact, with no reduction in muscle strength, and sensation in the saddle and perineal regions was preserved. Bowel and bladder function remained normal, with no urinary retention, urinary incontinence, or defecatory dysfunction. No perioperative surgery-related complications occurred, including new neurological injury, cerebrospinal fluid leakage, postoperative hematoma, or wound-related complications.The primary surgical objectives were complete tumor excision, relief of spinal cord compression, and maintenanceof spinal stability. Intraoperatively, the tumor exhibited an intact capsule, and gross-total resection was achieved. Histopathological examination confirmed the diagnosis of ependymoma (World Health Organization grade2). Although IDEM tumors often demonstrate benign imaging features, their potential for local invasiveness warrants caution. Complete surgical resection remains the cornerstone for preventing recurrence; radiotherapy is reserved exclusively for cases with subtotal resection or residual disease (2).

For patients with low-grade intracranial ependymoma who have undergone gross-total resection, long-term radiological surveillance is recommended over adjuvant therapy;this management strategy aligns with the indolent biological behavior of these tumors and is supported by clinical evidence (3). Moreover, intraoperative pedicle screw fixation can effectively reduce the risk of postoperative decline in spinal stability (4). IDEM ependymomas, although posing diagnostic challenges preoperatively—particularly in differentiating them from other Intradural space-occupying lesions—exhibit characteristic features including a slow clinical progression and an asymmetric anatomical location (5). For suspected intraspinal space-occupying lesions, contrast-enhanced MRI is recommended as the first-line imaging modality to evaluate tumor vascularity and its anatomical relationship with surrounding structures; neurophysiological studies may be added as needed. In cases presenting with neurological compression symptoms, surgicalresection is the treatment of choice. Intradural–extramedullary (IDEM) ependymomas account for approximately 3%–6% of all malignant central nervous system tumors (6). IDEM ependymomas, although less common than intracranial ependymomas, are associated with a relatively favorable prognosis. Adjuvant radiotherapy is recommended for WHO grade 3 tumors or for incompletely resected WHO grade 2 tumors (7). The main reason for not undergoing chemotherapy lies in the fact that low-grade intracranial and subdural extramedullary ependymomas have an inert biological behavior. Surgical resection usually leads to a good prognosis. Chemotherapy has limited efficacy and lacks evidence to support it. Adjuvant therapy mainly relies on radiotherapy for high-risk or partially resected cases.In cases of tumor recurrence, resistance to conventional therapy, or clinically significant radiation-related toxicity, emerging therapeutic options may include tyrosine kinase inhibitors, telomerase-targeted agents, anti-angiogenic therapies, and immunotherapeutic approaches (8).

If tumor recurrence, radiation-induced cognitive deficits, or chemotherapy resistance occur, therapeutic options may include tyrosine kinase inhibitors, telomerase inhibitors, anti-angiogenic agents, and immunotherapy (8). Genome sequencing has advanced the development of precision oncology; however, the oncogenic drivers of many malignant tumors remain elusive or lack well-defined therapeutic targets, necessitating alternative strategies for target identification. Take ependymoma as an example: this chemotherapy-resistant brain tumor has undergone extensive genomic sequencing analyses, yet effective therapeutic targets remain scarce (9). A recent survey indicated that, in cases with a lower cumulativenumber of tumor-affected spinal segments, minimally invasive surgery achieves tumor resection rates comparable to those of open surgery, significantly reduces the incidence of postoperative complications, effectively lowers perioperative healthcare costs, and shortens hospital stay. However, open surgery enables minimization of neurological injury (10). This case involved more than one spinal segment; given the preoperative MRI findings regarding the spatial relationship between the tumor and adjacent neurovascular structures, an open surgical approach was selected to optimize neural functional preservation and enable complete tumor resection. Spinal ependymomas comprise three molecular subtypes: spinal ependymoma (SP-EPN), myxopapillary ependymoma (SP-MPE), and subependymoma (SP-SE).SP-EPN and SP-MYCN must be diagnosed in relation to their specific spinal location, whereas SP-MPE and SP-SE can occur at other anatomical sites. All spinal ependymoma subtypes show a predilection for adult patients (11). Patients with rare central nervous system (CNS) tumors frequently confront poor prognoses and a paucity of effective therapeutic options.

A recent study has elucidated the molecular oncogenic mechanisms underlying a subset of these rare tumors, thereby motivating the development of high-fidelity, disease-representative preclinical models. Such models are indispensable for advancing targeted therapy research and hold promise for shifting the current treatment paradigm-largely reliant on conventional cytotoxic agents-toward more precise, mechanism-based interventions (12). Ependymomas originate from radial glial cells. Elongated-cell ependymoma-the most common subtype in the spinal cord-is characterized by fascicular architecture with variable width and cellular density, composed of slender, spindle-shaped cells. Grading of ependymomas remains a challenging and unresolved issue; given the established prognostic significance of molecular subgroups, treatment decisions should not rely solely on histopathological grade. SP-MPE is most commonly associated with aneuploidy or tetraploidy and exhibits dysregulated cellular metabolism, such as upregulation of the hypoxia pathway via HIF1α and increased reliance on glycolysis. SP-SE tumors have been found to harbor deletions of chromosome 6q (13). miRNAs serve as molecular biomarkers for clinical therapeutics; the CYP11B1 and KRT33B genes are involved in the estrogen biosynthesis signaling pathway, whereas UNX1T1 and SIK are associated with cancer development and tumor suppression processes (14). MYCN is a member of the oncogene family and has been implicated in the pathogenesis of various malignancies, including neuroblastoma, pediatric glioblastoma, and medulloblastoma, as well as leukemia and prostate cancer. To date, no clinical trials targeting MYCN have been initiated. However, several studies have evaluated MYCN-targeted inhibition strategies. These primarily include inhibitors targeting histone deacetylases (HDACs), poly(ADP-ribose) polymerase (PARP), aurora kinase A (AURKA), and bromodomain and extraterminal (BET) domain proteins, as well as DNA vaccine–based immunotherapies (15).

In a study, the treatment of central nervous system (CNS) malignancies faces numerous challenges, including the blood-brain barrier (BBB), tumor heterogeneity and high invasiveness, limited tissue accessibility for early diagnosis and effective surgical resection, and acquired resistance to anticancer therapies (16). Recently, lipid-based nanocarriers have been loaded with apoptosis- or ferroptosis-inducing agents, demonstrating appreciable antitumor efficacy (16). Addressing care barriers for patients with ependymoma necessitates a multidisciplinary collaborative approach that bridges pediatric and adult care paradigms, integrated with advanced molecular diagnostics and targeted therapeutics (17). This case was ultimately diagnosed pathologically as an intradural extramedullary (IDEM) ependymoma, based on the integration of preoperative contrast-enhanced lumbar spine MRI with postoperative immunohistochemical and histopathological findings. This diagnostic pathway underscores the critical importance of multimodal assessment—particularly the synergistic use of contrast-enhanced MRI and histopathological examination-in enhancing diagnostic accuracy and improving clinical recognition of atypical IDEM ependymomas, thereby preventing diagnostic delay attributable to unusual anatomical location. Moreover, it confirms the pivotal role of multimodal evaluation-especially the combination of contrast-enhanced MRI and pathology-in differentiating IDEM ependymomas, offering a novel perspective for the differential diagnosis of low back pain etiologies in adolescents and young adults. However, several limitations warrant acknowledgment: the single-case nature of the study limits generalizability, and the absence of long-term follow-up data (e.g.5-year recurrence rate) precludes robust assessment of postoperative oncologic outcomes. In summary, this report presents a rare case of an intradural extramedullary (IDEM) ependymoma arising at the atypical lumbar spinal levels L1–L3.It underscores the necessity to transcend the conventional diagnostic bias that ependymomas predominantly occur in the filum terminale-despite their well-established predilection for this region.

The current case, located in the upper lumbar spine (L1–L3), highlights the importance of broadening the differential diagnosis to encompass the entire spinal canal. Furthermore, it reaffirms the gold-standard role of integrated multimodal imaging and histopathological evaluation in the accurate diagnosis of such atypical presentations.For instance, contrast-enhanced MRI plays a pivotal role: key imaging features requiring careful evaluation include the relationship between the tumor and the dura mater, the pattern of contrast enhancement, and the morphology of spinal cord compression. Intradural ependymomas (IDEMs) typically manifest as well-circumscribed, capsule-like enhancing lesions on MRI; however, they may be radiologically indistinguishable from schwannomas (exhibiting the “target sign”) or meningiomas (showing the “dural tail sign”). Therefore, supplementary molecular pathological classification is essential. During surgical intervention, preservation of spinal cord neurological function and maintenance of spinal column stability must be prioritized. Multidisciplinary team (MDT) collaboration represents a critically important diagnostic approach-particularly for diseases characterized by rarity, anatomical heterogeneity, and subtle or nonspecific imaging findings. Its value is especially evident in overcoming diagnostic blind spots associated with atypical tumor locations. We therefore recommend that all cases of intradural extramedullary (IDEM) ependymoma be systematically incorporated into an MDT workflow to reduce misdiagnosis rates and improve both overall survival and health-related quality of life. This case report was prepared in accordance with the CARE (CAse REport) guidelines for reporting clinical cases (18).

## Data Availability

The original contributions presented in the study are included in the article/supplementary material. Further inquiries can be directed to the corresponding author.
